# Detection of Low-Frequency Mutations and Identification of Heat-Induced Artifactual Mutations Using Duplex Sequencing

**DOI:** 10.3390/ijms20010199

**Published:** 2019-01-08

**Authors:** Eun Hyun Ahn, Seung Hyuk Lee

**Affiliations:** 1 Department of Pathology, University of Washington, Seattle, WA 98195, USA; lee0504@uw.edu; 2 Institute of Stem Cell and Regenerative Medicine, University of Washington, Seattle, WA 98109, USA

**Keywords:** duplex sequencing, duplex consensus sequence (DCS), single strand consensus sequence (SSCS), next-Generation sequencing (NGS), sequencing error, rare mutations, oxidative DNA damage, heat-induced mutations, mitochondrial dna, human breast cells

## Abstract

We present a genome-wide comparative and comprehensive analysis of three different sequencing methods (conventional next generation sequencing (NGS), tag-based single strand sequencing (e.g., SSCS), and Duplex Sequencing for investigating mitochondrial mutations in human breast epithelial cells. Duplex Sequencing produces a single strand consensus sequence (SSCS) and a duplex consensus sequence (DCS) analysis, respectively. Our study validates that although high-frequency mutations are detectable by all the three sequencing methods with the similar accuracy and reproducibility, rare (low-frequency) mutations are not accurately detectable by NGS and SSCS. Even with conservative bioinformatical modification to overcome the high error rate of NGS, the NGS frequency of rare mutations is 7.0 × 10^−4^. The frequency is reduced to 1.3 × 10^−4^ with SSCS and is further reduced to 1.0 × 10^−5^ using DCS. Rare mutation context spectra obtained from NGS significantly vary across independent experiments, and it is not possible to identify a dominant mutation context. In contrast, rare mutation context spectra are consistently similar in all independent DCS experiments. We have systematically identified heat-induced artifactual variants and corrected the artifacts using Duplex Sequencing. Specific sequence contexts were analyzed to examine the effects of neighboring bases on the accumulation of heat-induced artifactual variants. All of these artifacts are stochastically occurring rare mutations. C > A/G > T, a signature of oxidative damage, is the most increased (170-fold) heat-induced artifactual mutation type. Our results strongly support the claim that Duplex Sequencing accurately detects low-frequency mutations and identifies and corrects artifactual mutations introduced by heating during DNA preparation.

## 1. Introduction

Next-generation sequencing (NGS) has rapidly transformed entire areas of basic research and therapeutic applications by making large scale genomic studies feasible through reduced cost and faster turnaround time [1,2]. Conventional NGS has been extensively used to study clonal (high-frequency) mutations, but not subclonal (low-frequency) mutations. A major impediment in investigating subclonal (low-frequency) mutations is that conventional NGS methods have high error rates (10^−2^ to 10^−3^), which obscure true mutations that occur less frequently than errors [3,4]. These subclonal mutations may account for the genetic heterogeneity of tumors and tumor recurrence, as well as provide a reservoir for the rapid development of resistance to chemotherapy [5].

Conventional sequencing technologies sequence only a single strand of DNA. In contrast, Duplex Sequencing examines both strands of DNA and scores mutations only if they are present on both strands of the same DNA molecule as complementary substitutions. This significantly reduces sequencing error rates to <5 × 10^−8^ [6,7,8,9]. In the first report of Duplex Sequencing, accuracy and sensitivity of mutation detection were demonstrated mainly in M13mp2 bacteriophage by comparing untreated/control DNA and DNA incubated with hydrogen peroxide, a radical generator, in the presence of iron [6].

While overall frequencies and types of mutations from conventional NGS, SSCS, and Duplex Sequencing have been compared in previous studies [5,10], those studies focused on detection limits of low-frequency mutations only and did not compare the sequencing methods’ ability to detect high-frequency mutations. In addition, influences of neighboring nucleotide base context on mutations (mutation context spectra) have not been investigated.

In the current study, we systematically compared mutation frequencies, types, positions, and sequence context spectra of the whole mitochondrial (mt) DNA in human breast epithelial cells using three different sequencing protocols: conventional NGS, tag-based single strand consensus sequencing (e.g., SSCS), and Duplex Sequencing. We applied the three sequencing methods to categorize and analyze high-frequency and low-frequency mutations, separately. Furthermore, analyses were done with several independent DNA library preparation experiments of an identical biological sample to evaluate the detection consistency, reproducibility, and validity of each sequencing method. Heating samples, a common practice in preparing DNA for molecular biology experiments, can introduce such artifactual mutations [11]. Herein, we present heat-induced artifactual mutations identified using Duplex Sequencing and specific nucleotide contexts that contribute to a high level of heat-induced artifactual mutations.

## 2. Results

Duplex Sequencing generates both SSCS and DCS analysis results. In Duplex Sequencing, both strands of DNA are individually tagged and strands with identical tag sequence, the product of the same DNA template, are grouped together after PCR amplification. SSCS analysis differs from DCS analysis in that complementary tag sequences are not identified, and so complementary strands are not grouped [6]. The SSCS method represents a tag-based single strand sequencing procedure and is comparable to that of Safe Sequencing System (SafeSeqS) in that each single-stranded DNA molecule is uniquely labeled before PCR amplification, allowing strands of the same derivatives to be grouped [9,12].

The average number of nucleotides sequenced at each genome position (depth) of all conventional NGS, SSCS, and DCS analyses were calculated as the total number of nucleotides sequenced divided by the mtDNA size of 16,569 bases. The average depths for conventional NGS, SSCS, and DCS analyses for normal human breast cells and immortalized cells are presented in Tables S1 and S2. The highest depths of conventional NGS, SSCS and DCS that were processed under the same data processing conditions were 458441, 40421 and 6803, respectively (Table S1).

**Figure 1:**
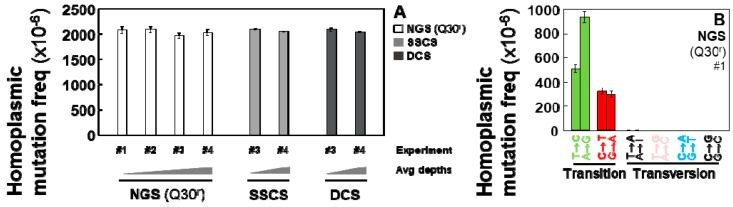
Frequencies of homoplasmic point mutations in the whole mtDNA of immortalized human breast cells. Error bars represent the Wilson score 95% confidence intervals. (**A**) Overall frequencies determined by performing conventional NGS, SSCS, and DCS analyses; (**B**) Homoplasmic mutation frequency of specific mutation types determined using conventional NGS (representative data). Same pattern of frequencies of specific mutation types is observed for all experiments regardless of the sequence method used.

**Figure 2:**
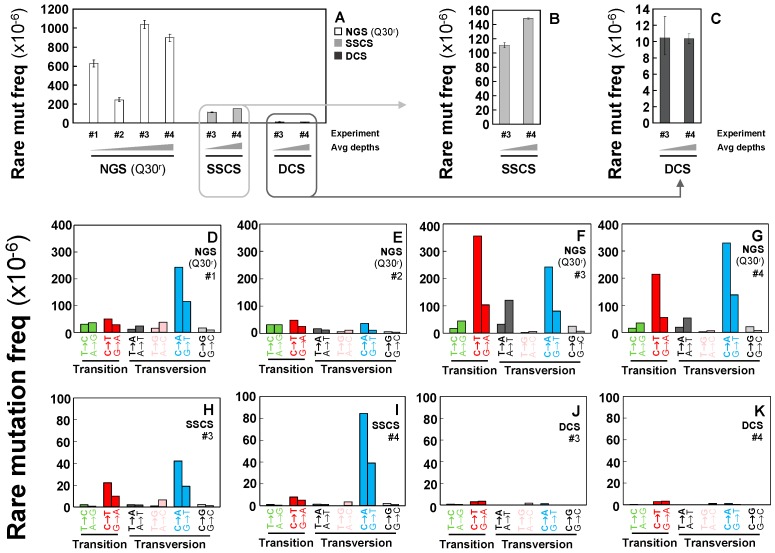
Frequencies of rare point mutations in the whole mtDNA of immortalized human breast cells. Error bars represent the Wilson score 95% confidence intervals; (**A**–**C**) Overall frequencies determined by performing conventional NGS, SSCS, and DCS analyses. Rare mutation frequency of each mutation type as determined using (**D**–**G**) conventional NGS, (**H**,**I**) SSCS, and (**J**,**K**) DCS analyses. Error bars represent the Wilson score 95% confidence intervals.

**Figure 3:**
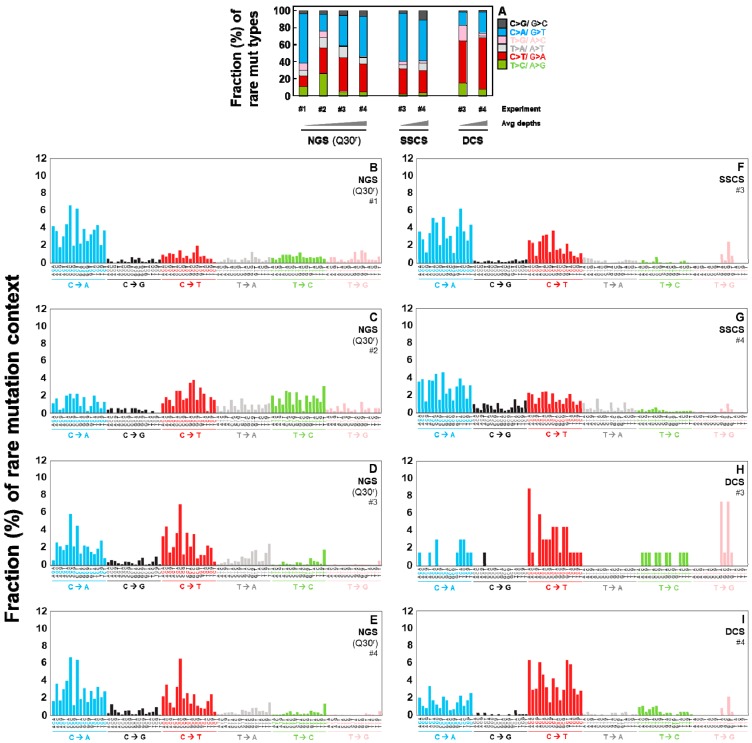
Fractions (%) of rare mutation types and context spectra in the whole mtDNA of immortalized human breast cells. Relative percentages (%) of mutation types (**A**) and fractions of rare mutation context spectra (**B–I**) were determined by performing conventional NGS, SSCS, and DCS analyses.

As an attempt to overcome the high error rates of conventional NGS, more conservative bioinformatical conditions than those applied for SSCS and DCS, referred to as conventional NGS (Q30^r^) hereinafter (See Section 4.4.2. Materials and Methods), were applied to conventional NGS datasets. Results of conventional NGS (the same bioinformatical conditions as to SSCS and DCS) and conventional NGS (Q30^r^) are presented in Supplementary Materials Section, Figures S1–S4. Figure 1, Figure 2 and Figure 3 compare the results of conventional NGS (Q30^r^) with those of SSCS and DCS.

For this study, we have defined homoplasmic (90–100%: Figure 1, Figures S1 and S2, Table S3) and rare (0–1%: Figure 2, Figure 3, Figure 4 and Figure 5, Figures S3–S6, Tables S1, S2 and S4–S7) mutations based on the mutation occurrence (%) at each genome position. Mutation frequencies were calculated by dividing the number of variants by the total number of nucleotides sequenced.

### 2.1. Homoplasmic Mutations are Detectable by All Three Methods (Conventional NGS, Tag-Based Single Strand Sequencing, and Duplex Sequencing) with the Similar Accuracy and Reproducibility

The overall frequencies (Figure 1A) of homoplasmic point mutations and frequencies of each mutation type (Figure 1B) are almost identical across all independent experiments of conventional NGS (Q30^r^), SSCS, and DCS analyses.

In our study, 35 identical homoplasmic unique mutations were detected in all independent experiments regardless of sequencing methods used (Table S3). Taken together, all three sequencing methods are accurate enough to study highly prevalent mutations such as germline mutations of the nuclear genome and homoplasmic mutations of the mitochondrial genome.

### 2.2. Rarely Occurring Mutations are Neither Accurately Detectable by Conventional NGS Methods nor Tag-Based Single Strand DNA Sequencing, but are Accurately Detectable by Duplex Sequencing

Rare mutation frequencies of immortalized human breast cells were determined using conventional NGS, SSCS, and DCS methods. The average rare mutation frequencies of the independent experiments are significantly lower in SSCS (1.30 × 10^−4^) and DCS (1.04 × 10^−5^) by 5-fold and 67-fold, respectively than that of conventional NGS (Q30^r^) (7.00 × 10^−4^) (Figure 2A, Table S1). This indicates that Duplex Sequencing removes false-positive artifactual mutations and significantly reduces the rare mutation frequencies.

The frequencies of rare mutations are highly variable in independent experiments analyzed with conventional NGS (Q30^r^) (Figure 2A), whereas frequencies of rare mutations show reproducible results in independent experiments of DCS of Duplex Sequencing (Figure 2A,C). It is noted that conventional NGS (Q30^r^) datasets were processed under more conservative conditions (See Section 4.4.2. Materials and Methods) than those of SSCS and DCS; however, these bioinformatical modifications only lowered rare mutation frequency by, on average, 35% (Figures S3, S4, and Table S1). Furthermore, variations in rare mutation frequencies are inconsistent between “the conventional NGS results with the bioinformatical default conditions” and “the conventional NGS results with the more conservative bioinformatical modification (Q30^r^)” across the four independent experiments. Before applying more conservative conditions of the bioinformatical modifications, the rare mutation frequencies of experiments #2 and #4 are significantly greater than those of experiments #1 and #3 respectively (Figure S3). Moreover, the frequencies are more comparable for experiments #1 and #2 compared to #3 and #4 (Figure S3). However, after the application of bioinformatical modification (Q30^r^), the rare mutation frequencies of experiments #1 and #3 are significantly greater than those of experiments #2 and #4, respectively and the frequencies of experiments #3 and #4 are comparable (Figure S3). This indicates that the bioinformatical modification alone is not possible to overcome the high error rate of NGS.

Frequencies of each type of rare mutations reveal other significant differences between conventional NGS (Q30^r^), SSCS, and DCS analyses. In conventional NGS (Q30^r^) results, C > T/G > A transitions and C > A/G > T transversions are identified at high frequencies (Figure 2D–G). In SSCS results, C > A/G > T transversions are the most predominant mutation type followed by C > T/G > A transitions (Figure 2H,I). In contrast, DCS results indicate that C > T/G > A transitions and C > A/G > T transversions are no longer predominant and no particular type is more prominent than others (Figure 2J,K). Our data suggest that C > A/G > T transversions appear to be the most prevalent type of artifactual variants that are scored by both conventional NGS (Q30^r^) (Figure 2D–G) and SSCS (Figure 2H,I) methods.

Proportions (%) of each type of rare mutations were analyzed. The prevalent rare mutation types differ under the three sequencing methods. C > A/G > T transversions are the most dominant type of rare mutation with conventional NGS (Q30^r^) data (Figure 3A); however, the fraction (%) of C > A/G > T transversions vary widely across the four independent conventional NGS experiments. In contrast, comparable fractions (%) of each mutation type are observed in both DCS independent experiments (Figure 3A).

Influences of neighboring bases on mutations were examined by conducting a mutation context spectra analysis. This analysis identifies bases immediately 5′ and 3′ to a mutated base (i.e., the mutation appears at the second position of each trinucleotide) and enables classifying observed substitutions into 96 categories (4 bases × 6 substitutions × 4 bases) [13,14]. Significant variations are observed for rare mutation context spectra of conventional NGS (Q30^r^) data across four independent experiments (Figure 3B–E) and it is not possible to identify a dominant mutation context among them. In contrast, rare mutation context spectra are similar in all independent DCS experiments. For example, C > T transitions in contexts ACA and ACT occur at persistently high proportions in DCS data (Figure 3H,I).

### 2.3. Duplex Sequencing Identifies and Corrects the Heat-Induced Artifactual Variants Introduced During DNA Sample Preparation

We investigated which specific types of artifactual variants are introduced during DNA sample preparation such as heat treatments and to what extent these artifacts can be corrected by Duplex Sequencing. DNA was isolated from normal human breast primary cells (II) and an aliquot of DNA was incubated at 65 °C for 9 h. Unheated DNA served as the control. Libraries of heated and control DNA were prepared for Duplex Sequencing. To identify heat-induced specific variant types, we performed both SSCS and DCS analyses. The average SSCS and DCS depths of the whole mtDNA genome were similar for control DNA and heated DNA: SSCS (control: 12,257 and heated: 11,622) and DCS (control 2248 and heated: 2510) (Table S2). To closely examine the heat-induced artifactual variants, which are not detectable or distinguishable by conventional sequencing methods, we investigated the rare variants that occur at a frequency of 1% or less using Duplex Sequencing.

**Figure 4:**
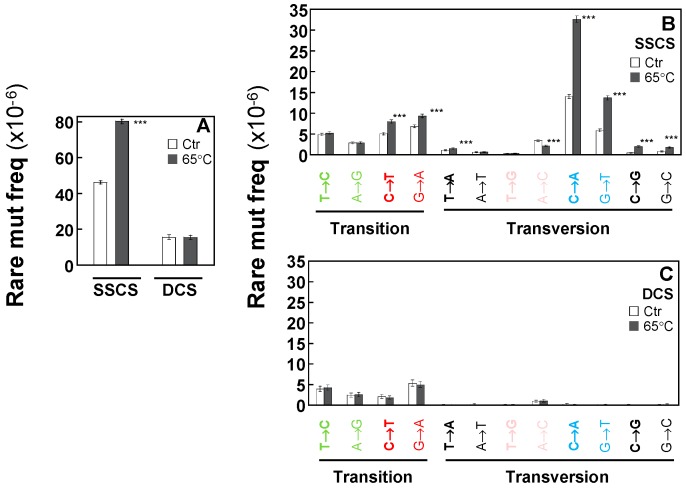
Frequencies of the heat-induced (65 °C) artifactual variants in normal human breast cells. Overall rare mutation frequency (**A**) and frequencies of rare mutation types (**B**,**C**) for heated versus control DNA were determined by SSCS and DCS analyses. Error bars represent the Wilson score 95% confidence intervals. The significant differences in rare mutation frequencies between the control DNA and the heated DNA are indicated (*** *p* < 5 × 10^−5^ by the Chi-square test).

The rare mutation frequencies of SSCS are significantly higher than those of DCS (Figure 4A). This higher SSCS mutation frequency could be due to heat-induced DNA damage and/or errors during PCR-amplification. Such artifactual variants are present on only one of the two DNA strands and thus they are not scored in DCS of Duplex Sequencing. While the incubation of DNA at 65 °C significantly increased the rare mutation frequency in SSCS analysis (Figure 4A: the first and second bars: *p*-value < 2.2 × 10^−16^), both heated and control DNA displayed identical frequencies of rare mutations in DCS analysis (Figure 4A: the third and fourth bars). Our results clearly indicate that DCS analysis by Duplex Sequencing is not affected by heat-induced DNA damage introduced during DNA sample preparation and correctly represents true mutations. 

### 2.4. Duplex Sequencing Identifies the Specific Mutation Spectra of Heat-Induced Artifacts 

We further examined which specific variant type(s) contributed to the elevated SSCS rare mutation frequency in heated DNA. In SSCS, but not DCS, the heated DNA (Figure 4B) shows a significant increase in rare variant frequencies of C > A/G > T, C > T/G > A, C > G/G > C versus control DNA (Figure 4B). In contrast, the 65 °C incubation (heating DNA) did not affect the rare mutation spectra of DCS results (Figure 4C). For example, the SSCS rare variant frequency of C > A in heated DNA is 3.26 × 10^−5^. This heat-induced artifactual variant type is significantly reduced by 170-fold to 1.88 × 10^−7^ in DCS analysis.

**Figure 5:**
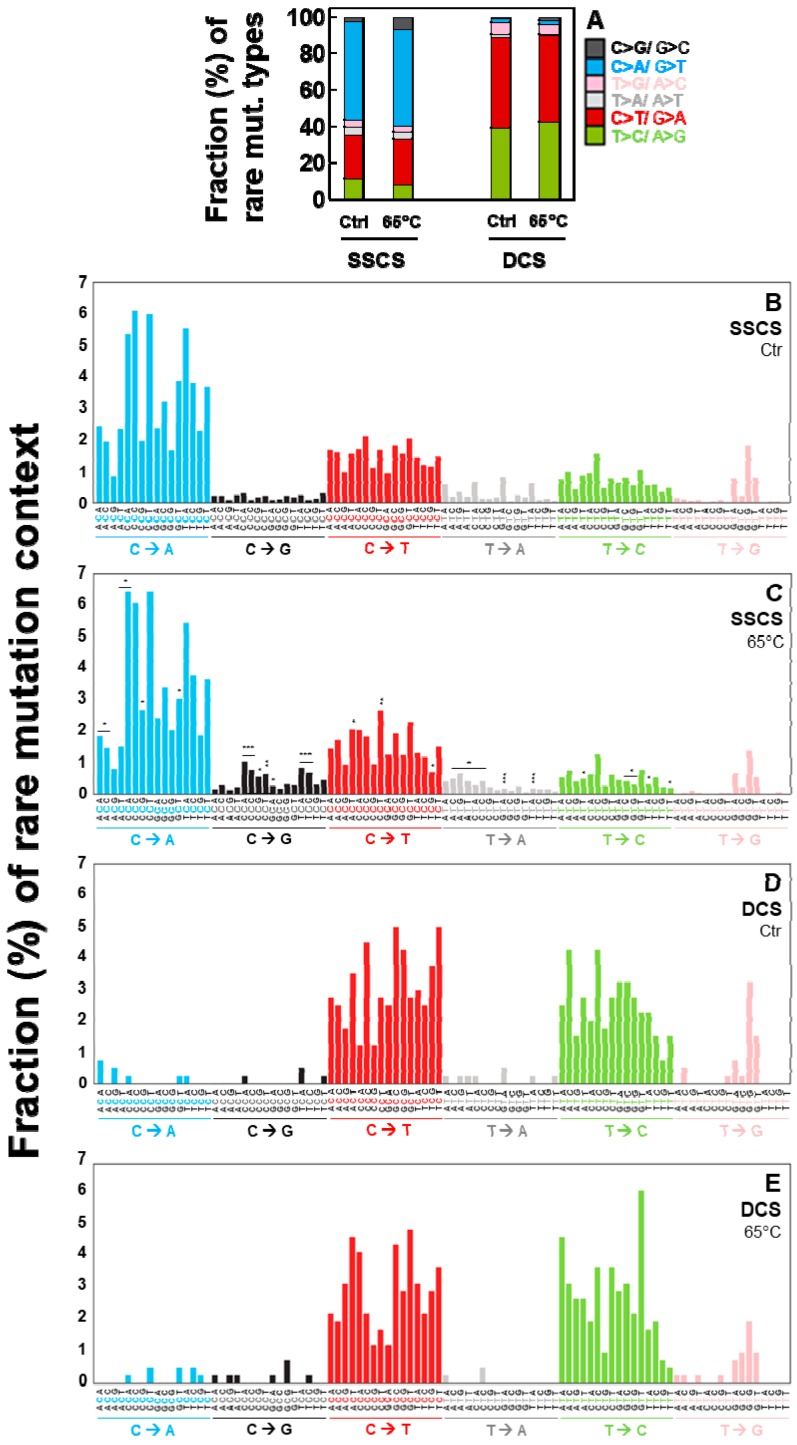
Fractions (%) of the heat-induced (65 °C) artifactual mutation types and context spectra in normal human breast cells. Relative percentages of rare mutation types (**A**) and rare mutation context spectra (**B**–**E**) for heated versus control DNA were determined by SSCS and DCS analyses. The significant differences in percentage of each mutation context between the control untreated DNA and the heated DNA under SSCS analyses (**C**) are indicated (* *p* < 0.05, ** *p* < 5 × 10^−4^, *** *p* < 5 × 10^−5^ by the Chi-square test).

Fractions (%) of each type (Figure 5A) and each context spectrum of rare mutations (Figure 5B–E) were examined for heated versus control DNA. The heat-induced DNA damage results in increases in C > G/G > C in SSCS analysis (gray-black bars in Figure 5A–C). Out of the 96 possible mutation sequence contexts, 28 are significantly changed after the 65 °C incubation in SSCS analysis (Figure 5B–C, Table S4). Particularly, CCC, TCC and CCA contexts of C > G mutations showed the most significant increase in the heated DNA compared to the control (unheated) DNA in SSCS analysis (Figure 5C, Table S4). In contrast, these DNA damage-dependent changes of specific mutation context spectra are not observed in DCS analysis, irrespective of neighboring nucleotides (Figure 5A,D,E).

### 2.5. Independent Experiments of Duplex Sequencing Reproducibly Identify the Heat-Induced Artifactual Mutaiton Profiles

Two independent cell culture and DNA library experiments (I and II) for the incubation of DNA at 65 °C for 9 h were conducted with DNA isolated from normal human epithelial cells (I and II) derived from breast tissue of the same woman. The average SSCS and DCS depths were similar between the cells I and cells II: SSCS (I: 11,622 and II: 11,045) and DCS (I: 2510 and II: 2460) (Table S5).

Rare mutation frequencies of heated DNA of the cells I (Figure S5) were calculated for both SSCS and DCS, and the analysis reveals the same pattern observed with heated DNA of the cells II used for the main result Figure 4 experiment (Figure S5). Both the overall rare mutation frequencies and the rare mutation frequency of each mutation type are observed at similar levels between the heated DNA of the cells I and II (Figure S5). Furthermore, fractions (%) of each type (Figure S6A) and context spectra (Figure S6B–E) of rare mutations are almost identical, strengthening the finding that Duplex Sequencing is capable of identifying and correcting the heat-induced artifactual mutations and the results are reproducible in independent experiments.

### 2.6. All Identified Heat-Induced Artifactual Variants are Stochastically Occurring Variants Throughout the Whole Mitochondrial Genome

**Figure 6:**
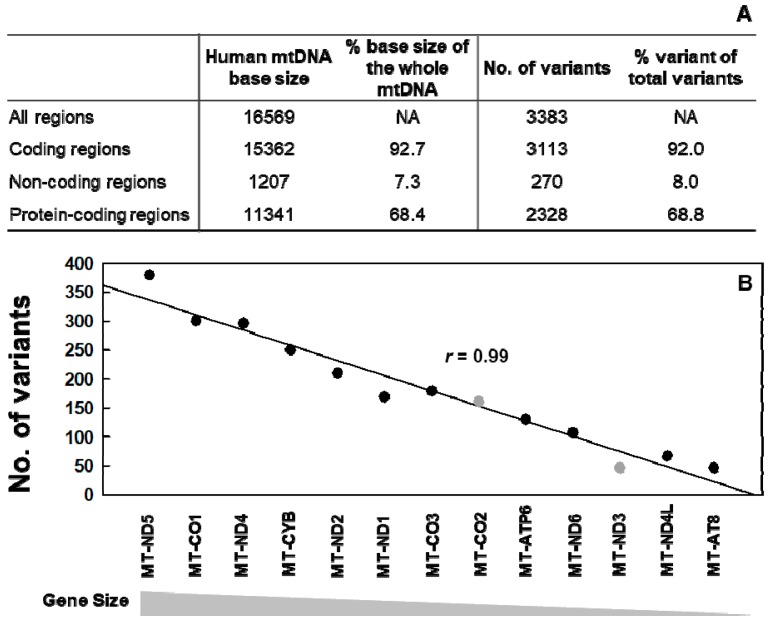
Numbers of the heat-induced (65 °C) artifactual variants in the whole mtDNA of normal human breast cells identified by SSCS and DCS analyses. Variants were counted only once at each position of the genome. (**A**) Numbers of variants identified in coding, protein-coding, and non-coding regions of mtDNA. The percentage of variants from each region out of the total number of identified variants is calculated (% variants). The percentage of base size that each region occupies out of the total size of mtDNA is calculated (% base size); (**B**) The numbers of the heat-induced artifactual rare variants identified in each of 13 protein-coding genes of mtDNA are plotted in the order of largest to smallest gene size. The Pearson’s correlation coefficient for each gene size of the 13 genes versus numbers of variants of each gene was 0.99 (*p* = 2.36 × 10^−10^).

A total of 3383 heat-induced artifactual unique variants were identified, all these are in the variant occurrence (%) range of 0–1% (Figure 6A). This clearly indicates that all of the heat-induced artifactual variants introduced during DNA preparation are rarely occurring variants, thus not accurately and reliably detectable by conventional DNA sequencing methods.

Of the all identified artifactual variants, about 92% of them are found on coding regions of mtDNA and 69% of them are found within the 13 protein-coding regions of mtDNA (Figure 6A, Table S6). The percentages of artifactual variants found on coding regions and 13 protein-coding regions of mtDNA closely match the percentages these two regions occupy in the whole mtDNA, which are about 92% and 68%, respectively [15]. In addition, the number of artifactual variants identified shows a strong positive correlation with the sizes of 13 protein-coding genes (Pearson’s correlation coefficient *r* = 0.99 and *p* = 2.36 × 10^−10^), which indicates that more artifactual variants are found on larger genes (Figure 6B). We examined the 13 protein-coding genes of mtDNA to see if any particular gene is relatively more or less prone to heat-induced variants. For each gene, we calculated the percent of variants by dividing the numbers of variants by each gene size (bases). Among the 13 genes, MT-CO2 is slightly more mutated at 23.39% and MT-ND3 is mutated the least at 13.01% (Table S7). The majority (11 out of 13) of these genes are mutated at similar variant occurrences (average 20%), which is consistent with the finding that artifactual variants occur stochastically.

## 3. Discussion

In this study, we sequenced the entire mitochondrial DNA genome of human breast cells via three different sequencing protocols: conventional NGS, tag-based single strand sequencing (e.g., SSCS), and Duplex Sequencing. We systematically compared high-frequency and low-frequency mutations obtained from the three methods. We demonstrate advantages of Duplex Sequencing over other sequencing methods for studying rarely occurring mutations. In addition, we identified the heat-induced artifactual variants. Although Duplex Sequencing has been used in a previous study to show an increased level of mutation frequency of small selected regions of nuclear genome in DNA incubated at 65 °C [11], the same temperature used in this study, exact identities of the heat-induced artifactual variants have not been presented. Moreover, while types of artifactual variants have been previously examined, influences of neighboring nucleotide base context on artifactual variants have not been investigated. To our knowledge, this is the first study to present the exact identities of the heat-induced artifactual variants and the specific nucleotide context spectra for these artifacts.

Our data show that rare mutation frequencies are significantly lower in DCS analysis in comparison to conventional NGS and SSCS analyses, suggesting that large number of rare mutations detected by conventional NGS and SSCS are mostly artifacts (Figure 2A). Particularly, C > A/G > T transversions, which have been previously reported to be a predominant result of DNA oxidation [11,16], showed the greatest decrease in frequencies with DCS analyses of Duplex Sequencing (Figure 2J,K). These findings validate that DCS can identify and correct artifacts and be applied for accurately detecting rarely occurring mutations. Furthermore, the comparison of rare mutation frequencies between multiple independent experiments (Figure 2) demonstrates the ability of DCS in producing reproducible results. However, the rare mutation data by conventional NGS and SSCS shows high variability across independent experiments, which indicates the lack of the capability of conventional NGS and SSCS to produce reliable and reproducible results. The comparison of rare mutation context spectra analyses (Figure 3) further distinguishes DCS from conventional NGS and SSCS by showcasing its advantage to accurately and consistently detect rarely occurring mutations.

Artifactual variants can be generated as a result of copying damaged DNA bases. Such variants are present on only one of the two DNA strands and are scored by conventional NGS and SSCS but not by DCS. In the present study, we have identified heat-induced artifactual variants by performing both SSCS and DCS analyses. Our results indicate that C > A/G > T is the most predominantly enhanced artifactual variant followed by C > T/G > A and C > G/G > C. Previous studies reported that heating DNA can damage DNA bases by forming oxygen free radicals, (specifically 8-hydroxy-2′-deoxyguanine (8-Oxo-dG)), which deaminate cytosine to uracil, and increasing mitochondrial superoxide anion, which also leads to oxidation of DNA [17,18,19]. The 8-Oxo-dG is generated by DNA oxidation under physiopathological conditions or environmental stress. It is also a by-product of normal cellular metabolism [20]. The formation of 8-oxoguanine, particularly 8-oxo-dG has been reported to cause a high level of C > A/G > T mutations [11,16,20,21,22,23], whereas deamination of cytosine to uracil is known to produce high levels of C > T/G > A mutations [11,19]. In one study, conventional NGS analysis was done on tumors and matching normal tissues of melanoma and an enzyme-linked immunosorbent assay (ELISA) for 8-Oxo-dG found that the CCG > CAG context have a high potential for being a target of DNA oxidation [22]. This context is observed at high mutation frequencies (2-fold increase) in our current study. Thus, it is likely that the high prevalence of C > A/G > T transversions in our data is caused by 8-Oxo-dG, suggesting that the biggest contributor of heat-induced artifactual variants is oxidative damage to mtDNA.

To our knowledge, this study is the first to examine mutation context spectra of heat-induced artifactual variants in the whole mitochondrial DNA of human breast epithelial cells. Our SSCS analysis results showed that out of 96 possible mutation sequence contexts, the fraction of 28 rare mutation sequence contexts were significantly changed after the 65 °C incubation (Figure 5B,C, Table S4). Among the affected 28 mutation context spectra, CCC, TCC and CCA contexts of C > G variants showed the most significant increase. These mutation contexts could be more prone to DNA damage and may have a high potential for being targeted by molecular reactions which result from the heat-damaged DNA.

In summary, we present a genome-wide comprehensive and comparative analysis of mitochondrial DNA mutations for conventional NGS and tag-based methods (single-strand sequencing and Duplex Sequencing) and demonstrate the identification and removal of heat-induced artifactual variants using Duplex Sequencing. Our results indicate that all of the heat-induced artifactual variants are stochastically occurring rare variants. Thus, these artifactual variants are not accurately detectable by conventional sequencing methods. Even the application of more conservative bioinformatical modification on conventional NGS datasets is not enough to overcome the inherently high error rate of conventional NGS methods. Our data establishes that Duplex Sequencing: (1) accurately and reproducibly detect rare (low-frequency) mutations; (2) is not affected by damage introduced by heating during DNA preparation; (3) identifies and removes the DNA damage-induced artifactual variants.

## 4. Materials and Methods

### 4.1. Cell Culture

Human breast epithelial cells were provided by Drs. Chia-Cheng Chang at Michigan State University in East Lansing, MI, USA. Normal human breast cells used in this study were isolated from breast tissues of a healthy (cancer-free) women 21–29 years of age obtained during reduction mammoplasty at Sparrow Hospital in Lansing, MI, USA. The donors were given ID #13 and #31 for the cells used in this study. Immortalized human breast cells (M13SV1) used for the experiments comparing the three sequencing methods were derived from the parental normal stem cells of woman ID #13 by transforming with SV40 T antigen [24,25,26,27,28,29]. The normal cells used for the heat-induced DNA damage experiments were from woman ID #31. Written consents were received from patients. The use of human breast cells was approved by Michigan State University Institutional Review Board and a Material Transfer Agreement was approved by both Michigan State University and University of Washington. The cells were cultured as previously described [30,31] and were authenticated by short tandem repeat (STR) DNA profiling (Genetica DNA Laboratories, Labcorp brand, Burlington, NC, USA).

### 4.2. DNA Extraction, Adapter Synthesis, and Library Preparation

DNA extraction and purification, adapter synthesis, and DNA library preparation for Duplex Sequencing [7,31] and for whole exome sequencing (WES) [32,33] were carried out as described previously. DNA of human breast epithelial cells (immortalized cells: Figure 1, Figure 2 and Figure 3, Figures S1–S4, and Tables S1 and S3; normal cells: Figure 4, Figure 5 and Figure 6, Figures S5 and S6 and Tables S2 and S4–S7) was extracted using a commercially available DNA extraction kit (Invitrogen, Thermo Fisher Scientific Inc., Carlsbad, CA, USA) with a lysis buffer (10 mM Tris-HCl, pH 8.0, 150 mM NaCl, 20 mM EDTA, 1% SDS).

For Duplex Sequencing, the sheared DNA was subjected to end-repair with 3′-dT-tailing for adapters with A-overhang or with 3′-dA-tailing for adapter with T-overhang. Before the ligation of DNA with the adapters, DNA was quantitated using quantitative real-time RT-PCR. Then, twenty-fold molar excess of adapter relative to DNA was added for the ligation process and the whole mitochondrial genome was captured using Agilent SureSelect^XT^ target enrichment set protocol version 1.6 (Agilent Technologies Inc., Santa Clara, CA, USA). The DNA libraries were sequenced via Illumina HiSeq 2500 (Illumina Inc., San Diego, CA, USA). A comprehensive and complete list of all materials and equipment used for Duplex Sequencing experiments is available on page 2594 of the manuscript *Detecting ultralow-frequency mutations by Duplex Sequencing* by Kennedy et al. 2014 [7].

For WES, the extracted DNA was sonicated to approximately 200 bp fragments then used to make a library for paired-end sequencing on an Illumina HiSeq 2500 platform (Illumina Inc., San Diego, CA, USA). The DNA library was captured by SeqCap EZ Exome v2 (Roche NimbleGen Inc., Madison, WI, USA) pools and then was hybridized to biotinylated capture probes.

### 4.3. Heat-Induced DNA Damage 

Normal human breast epithelial cells were used for the heat-induced DNA damage experiments (Figure 4, Figure 5 and Figure 6, Figures S5 and S6). Before DNA library preparation, an aliquot of DNA was incubated at 65 °C for 9 h to induce DNA damage. Then, same experimental protocols were applied for both heated and unheated DNA as described in Section 4.2. Materials and Methods.

### 4.4. Data Processing

#### 4.4.1. Conventional NGS Datasets and conventional NGS, SSCS and DCS Data Processing

Two of four independent conventional NGS datasets (Experiments #1 and #2 in Figure 1 through Figure 3) were obtained by extracting the whole mitochondrial genome data from our WES results. WES is a commonly used next-generation sequencing method that is able to read the entire exome of DNA as well as the mitochondrial genome [34]. The fastq data files for the two WES datasets were processed as previously described [33] with some modifications. Our in-house script was modified to align the reads with the human mitochondria reference file and to include the mitochondrial genome only.

Two additional independent conventional NGS datasets were obtained by modifying our in-house Duplex Sequencing script to simulate conventional NGS processing on DNA libraries prepared for Duplex Sequencing (Experiments #3 and #4 in Figure 1 through Figure 3). The DNA libraries were prepared for Duplex Sequencing; however, the Duplex Sequencing script was modified to proceed only through alignment with a human mtDNA reference but to not do single strand consensus sequence (SSCS) and duplex consensus sequence (DCS) data alignment steps. This negates the individuality of complementary DNA strands when processing sequence data and produces bioinformatically simulated NGS data that is comparable to conventional NGS.

SSCS and DCS datasets were processed as described previously [31]. All datasets were aligned to the Revised Cambridge Reference sequence (rCRS) reference genome, sequence number NC_012920, using BWA and genome analysis toolkit (GATK) software as described previously [31].

#### 4.4.2. Base Quality and PCR Duplicates

An illumina^®^ base quality score of 30 (Q30) is considered the benchmark for a correct base call in NGS. This score refers to a 1 in 1000 chance of an incorrect base call (error probability of 0.1% versus error probability of 5% with the default score of 13) [35]. Our NGS (Q30^r^) datasets, therefore, were processed with the base quality filtering adjusted to 30 from the default value of 13 by adding “-Q30” to the pileup command (i.e., samtools mpileup -B -d 500,000 -Q30 -f [reference] [input] [output]). However, introduction of artifacts in early stages of PCR amplification are not detectable as errors and are embedded in multiple PCR duplicates [6]. PCR duplicates were removed for conventional NGS (Q30^r^) data by taking the combined sequence-1 and sequence-2 files in bam format and using the command “samtools rmdup –s” of SAMtools software (Genome Research Ltd., Hinxton, UK) [36]. Both bioinformatical modifications accommodate high background error rates of conventional NGS. For DCS and SSCS data analyses, the default base quality score of 13 (error probability of 5%) was used and PCR duplicate removal was not applied since SSCS and DCS analyses have significantly lower error frequencies (1 × 10^−5^ and <5 × 10^−8^ or 1 × 10^−8^, respectively) than that of conventional NGS (10^−2^ to 10^−3^) [4,9]. Another reason the PCR duplicate removal step is not needed for SSCS and DCS analyses is that the molecular tags mark the duplicates for SSCS and DCS analyses.

The results of conventional NGS analysis obtained under the same bioinformatical conditions as SSCS and DCS (Q13 and no PCR duplicate removal) were presented in Table S1. The effects of bioinformatical modifications (Q13 and no PCR duplicate removal versus Q30 and PCR duplicate removal) on conventional NGS mutation results are presented in Supplementary Materials, Figures S1–S4.

#### 4.4.3. Comparison of Variant Positions

To identify the heat-induced artifactual variants, variant positions were compared after removing the common variants (present in both control and heated DNA) identified by SSCS and DCS analyses. Then, variants found only in the heated DNA from SSCS analysis were considered as heat-induced artifactual variants. Only genome positions that had minimum sequence read (depth) of 20 in both samples were considered.

#### 4.4.4. Counting Mutations

For calculating mutation frequencies, total number of variant reads observed were divided by total number of sequenced reads. For all other analyses, including fractions (%) of each mutation type and mutation context spectra, mutants were scored only once at each position of the genome (i.e., mutants were counted as 1 for each position regardless of number of variant reads observed in that position).

### 4.5. Statistical Analysis

Differences in mutation frequencies and mutation context fractions between control and heated DNA were analyzed by performing a Chi-square test using R (program version 3.4.4). Association of the number of identified unique variants in each of the thirteen protein-coding genes of mtDNA and the sizes of the corresponding thirteen genes was analyzed by Pearson correlation using Sigma Plot (version 12.0, Systat Software, Inc., San Jose, CA, USA). Differences between the groups were considered significant at *p* < 0.05.

## Supplementary Materials

Supplementary materials can be found at http://www.mdpi.com/1422-0067/20/1/199/s1.

## Abbreviations

DCS	Duplex Consensus Sequence
DS	Duplex Sequencing
Mt	Mitochondrial
NGS	Next-generation Sequencing
SSCS	Single Strand Consensus Sequence

## References

[1] Wetterstrand K.A. (2013). DNA Sequencing Costs: Data from the NHGRI Genome Sequencing Program (GSP).

[2] Goodwin S., McPherson J.D., McCombie W.R. (2016). Coming of age: Ten years of next-generation sequencing technologies. Nat. Rev. Genet..

[3] Lou D.I., Hussmann J.A., McBee R.M., Acevedo A., Andino R., Press W.H., Sawyer S.L. (2013). High-throughput DNA sequencing errors are reduced by orders of magnitude using circle sequencing. Proc. Natl. Acad. Sci. USA.

[4] Fox E.J., Reid-Bayliss K.S., Emond M.J., Loeb L.A. (2014). Accuracy of Next Generation Sequencing Platforms. Next Gener. Seq. Appl..

[5] Loeb L.A. (2016). Human Cancers Express a Mutator Phenotype: Hypothesis, Origin, and Consequences. Cancer Res..

[6] Schmitt M.W., Kennedy S.R., Salk J.J., Fox E.J., Hiatt J.B., Loeb L.A. (2012). Detection of ultra-rare mutations by next-generation sequencing. Proc. Natl. Acad. Sci. USA.

[7] Kennedy S.R., Schmitt M.W., Fox E.J., Kohrn B.F., Salk J.J., Ahn E.H., Prindle M.J., Kuong K.J., Shen J.-C., Risques R.-A. (2014). Detecting ultralow-frequency mutations by Duplex Sequencing. Nat. Protoc..

[8] Schmitt M.W., Fox E.J., Prindle M.J., Reid-Bayliss K.S., True L.D., Radich J.P., Loeb L.A. (2015). Sequencing small genomic targets with high efficiency and extreme accuracy. Nat. Methods.

[9] Salk J.J., Schmitt M.W., Loeb L.A. (2018). Enhancing the accuracy of next-generation sequencing for detecting rare and subclonal mutations. Nat. Rev. Genet..

[10] Newman A.M., Lovejoy A.F., Klass D.M., Kurtz D.M., Chabon J.J., Scherer F., Stehr H., Liu C.L., Bratman S.V., Say C. (2016). Integrated digital error suppression for improved detection of circulating tumor DNA. Nat. Biotechnol..

[11] Arbeithuber B., Makova K.D., Tiemann-Boege I. (2016). Artifactual mutations resulting from DNA lesions limit detection levels in ultrasensitive sequencing applications. DNA Res..

[12] Kinde I., Wu J., Papadopoulos N., Kinzler K.W., Vogelstein B. (2011). Detection and quantification of rare mutations with massively parallel sequencing. Proc. Natl. Acad. Sci. USA.

[13] Alexandrov L.B., Nik-Zainal S., Wedge D.C., Aparicio S.A.J.R., Behjati S., Biankin A.V., Bignell G.R., Bolli N., Borg A., Børresen-Dale A.-L. (2013). Signatures of mutational processes in human cancer. Nature.

[14] Pilati C., Shinde J., Alexandrov L.B., Assié G., André T., Hélias-Rodzewicz Z., Ducoudray R., Le Corre D., Zucman-Rossi J., Emile J.-F. (2017). Mutational signature analysis identifies *MUTYH* deficiency in colorectal cancers and adrenocortical carcinomas: Mutational signature associated with *MUTYH* deficiency in cancers. J. Pathol..

[15] MITOMAP A Human Mitochondrial Genome Database. http://www.mitomap.org/.

[16] Cheng K.C., Cahill D.S., Kasai H., Nishimura S., Loeb L.A. (1992). 8-Hydroxyguanine, an abundant form of oxidative DNA damage, causes G-T and A-C substitutions. J. Biol. Chem..

[17] Bruskov V.I., Malakhova L.V., Masalimov Z.K., Chernikov A.V. (2002). Heat-induced formation of reactive oxygen species and 8-oxoguanine, a biomarker of damage to DNA. Nucl. Acids Res..

[18] Slimen I.B., Najar T., Ghram A., Dabbebi H., Ben Mrad M., Abdrabbah M. (2014). Reactive oxygen species, heat stress and oxidative-induced mitochondrial damage. A review. Int. J. Hyperth..

[19] Kang Q., Parkin B., Giraldez M.D., Tewari M. (2016). Mutant DNA quantification by digital PCR can be confounded by heating during DNA fragmentation. BioTechniques.

[20] Cooke M.S., Evans M.D., Dizdaroglu M., Lunec J. (2003). Oxidative DNA damage: Mechanisms, mutation, and disease. FASEB J..

[21] Marnett L.J. (2000). Oxyradicals and DNA damage. Carcinogenesis.

[22] Costello M., Pugh T.J., Fennell T.J., Stewart C., Lichtenstein L., Meldrim J.C., Fostel J.L., Friedrich D.C., Perrin D., Dionne D. (2013). Discovery and characterization of artifactual mutations in deep coverage targeted capture sequencing data due to oxidative DNA damage during sample preparation. Nucl. Acids Res..

[23] Chen L., Liu P., Evans T.C., Ettwiller L.M. (2017). DNA damage is a pervasive cause of sequencing errors, directly confounding variant identification. Science.

[24] Kao C.Y., Nomata K., Oakley C.S., Welsch C.W., Chang C.C. (1995). Two types of normal human breast epithelial cells derived from reduction mammoplasty: Phenotypic characterization and response to SV40 transfection. Carcinogenesis.

[25] Kang K.S., Morita I., Cruz A., Jeon Y.J., Trosko J.E., Chang C.C. (1997). Expression of estrogen receptors in a normal human breast epithelial cell type with luminal and stem cell characteristics and its neoplastically transformed cell lines. Carcinogenesis.

[26] Chang C.C., Sun W., Cruz A., Saitoh M., Tai M.H., Trosko J.E. (2001). A human breast epithelial cell type with stem cell characteristics as target cells for carcinogenesis. Radiat. Res..

[27] Park J.-S., Noh D.-Y., Kim S.-H., Kim S.-H., Kong G., Chang C.-C., Lee Y.-S., Trosko J.E., Kang K.-S. (2004). Gene expression analysis in SV40-immortalized human breast luminal epithelial cells with stem cell characteristics using a cDNA microarray. Int. J. Oncol..

[28] Tai M.-H., Chang C.-C., Kiupel M., Webster J.D., Olson L.K., Trosko J.E. (2005). Oct4 expression in adult human stem cells: Evidence in support of the stem cell theory of carcinogenesis. Carcinogenesis.

[29] Ahn E.H., Chang C.-C., Schroeder J.J. (2006). Evaluation of sphinganine and sphingosine as human breast cancer chemotherapeutic and chemopreventive agents. Exp. Biol. Med..

[30] Ahn E.H., Lee S.H., Kim J.Y., Chang C.-C., Loeb L.A. (2016). Decreased Mitochondrial Mutagenesis during Transformation of Human Breast Stem Cells into Tumorigenic Cells. Cancer Res..

[31] Ahn E.H., Hirohata K., Kohrn B.F., Fox E.J., Chang C.-C., Loeb L.A. (2015). Detection of Ultra-Rare Mitochondrial Mutations in Breast Stem Cells by Duplex Sequencing. PLoS ONE.

[32] Walsh T., Shahin H., Elkan-Miller T., Lee M.K., Thornton A.M., Roeb W., Abu Rayyan A., Loulus S., Avraham K.B., King M.-C. (2010). Whole Exome Sequencing and Homozygosity Mapping Identify Mutation in the Cell Polarity Protein GPSM2 as the Cause of Nonsyndromic Hearing Loss DFNB82. Am. J. Hum. Genet..

[33] Gulsuner S., Walsh T., Watts A.C., Lee M.K., Thornton A.M., Casadei S., Rippey C., Shahin H., Consortium on the Genetics of Schizophrenia (COGS), PAARTNERS Study Group (2013). Spatial and temporal mapping of de novo mutations in schizophrenia to a fetal prefrontal cortical network. Cell.

[34] Griffin H.R., Pyle A., Blakely E.L., Alston C.L., Duff J., Hudson G., Horvath R., Wilson I.J., Santibanez-Koref M., Taylor R.W. (2014). Accurate mitochondrial DNA sequencing using off-target reads provides a single test to identify pathogenic point mutations. Genet. Med..

[35] Cliften P., Kulkarni S., Pfeifer J. (2015). Chapter 7: Base Calling, Read Mapping, and Coverage Analysis. Clinical Genomics.

[36] Li H., Handsaker B., Wysoker A., Fennell T., Ruan J., Homer N., Marth G., Abecasis G., Durbin R. (2009). 1000 Genome Project Data Processing Subgroup the Sequence Alignment/Map format and SAMtools. Bioinformatics.

