# Supplementary material for: Detection of Low-Frequency Mutations and Identification of Heat-Induced Artifactual Mutations Using Duplex Sequencing
Source: Int J Mol Sci. 2019 Jan 8;20(1):199. doi: 10.3390/ijms20010199 (PMC6337315; doi:10.3390/ijms20010199)
Supplement: Supplementary file 1 [file ijms-20-00199-s001.zip › Suppl_TableFig20190104.pdf]

Table S1. Sequencing data yield of human breast immortalized cells. The average depth (number of nucleotides sequenced at each genome position) was calculated as the total number of nucleotides sequenced divided by the mitochondrial (mt) DNA size of 16569 bases.

A. NGS and NGS (Q30^r) data yield (Data used for Figures 1-4 and S1-S4)

Exp	Avg NGS depth	Total sequenced NGS Nts	**NGS: No. of rare muts**	**NGS: Rare mut freq**	Avg NGS (Q30^r) depth	Total sequenced NGS (Q30^r) Nts	**NGS (Q30^r): No. of rare muts**	**NGS (Q30^r): Rare mut freq**
#1	517	8558610	**9803**	**1.15 $\times 10^{-3}$**	109	1810307	**1131**	**6.25 $\times 10^{-4}$**
#2	2130	35293720	**45526**	**1.29 $\times 10^{-3}$**	138	2279999	**556**	**2.44 $\times 10^{-4}$**
#3	210473	3487319586	**2476099**	**7.10 $\times 10^{-4}$**	140	2311906	**2395**	**1.04 $\times 10^{-3}$**
#4	458441	7595916093	**8773866**	**1.16 $\times 10^{-3}$**	140	2322756	**2084**	**8.97 $\times 10^{-4}$**

B. SSCS and DCS data yield (Data used for Figures 1-4 and S1-S4)

Exp	Avg SSCS depth	Total sequenced SSCS Nts	**SSCS: No. of rare muts**	**SSCS: Rare mut freq**	Avg DCS depth	Total sequenced DCS Nts	**DCS: No. of rare muts**	**DCS: Rare mut freq**
#3	2086	34562417	**3838**	**1.11 $\times 10^{-4}$**	444	7363685	**77**	**1.05 $\times 10^{-5}$**
#4	40421	669733398	**99361**	**1.48 $\times 10^{-4}$**	6803	112714571	**1164**	**1.03 $\times 10^{-5}$**

Abbreviations used are: Avg, average; Nts, nucleotides; mut, mutation; freq, frequency

Table S2. Sequencing data yield for heated *vs.* control DNA of human breast normal cells (II).

Cells	Avg SSCS depth	Total sequenced SSCS Nts	**SSCS: No. of rare muts**	**SSCS: Rare mut freq**	Avg DCS depth	Total sequenced DCS Nts	**DCS: No. of rare muts**	**DCS: Rare mut freq**
Control (Data for Figs 5, 6)	12257	203078000	**9351**	**4.60 $x10^{-5}$**	2248	37240836	**577**	**1.55 $x10^{-5}$**
Heated (Data for Figs 5, 6, S5, S6)	11622	192572847	**15429**	**8.01 $x10^{-5}$**	2510	41582269	**635**	**1.53 $x10^{-5}$**

Abbreviations used are: Avg, average; Nts, nucleotides; SSCS, single strand consensus sequences; DCS, duplex consensus sequences; mt, mitochondria; mut, mutation; freq, frequency

Table S3. Identical homoplasmic unique mutations detected using next-generation sequencing (NGS $Q30^{r}$), SSCS, and DCS analyses in all independent experiments for mtDNA of human breast immortalized cells. The mutations are listed in the order of largest to smallest gene size. Green represents T>C/A>G mutations and red represents C>T/G>A mutations.

Gene	DNA mutation	Amino acid mutations	Mutation type
MT-ND5	G12372A	*Syn*	
	A13117G	I261V	
MT-RNR2	A1811G	*Syn*	
	A2706G	N346D	
MT-CO1	G6179A	*Syn*	
	C6587T	*Syn*	
	C7028T	*Syn*	
MT-ND4	T11299C	*Syn*	
	A11362G	*Syn*	
	A11467G	*Syn*	
	G11719A	*Syn*	
MT-CYB	C14766T	T7I	
	T14798C	F18L	
	A15326G	T194A	
MT-ND1	A3480G	*Syn*	
	A4769G	*Syn*	
MT-RNR1	A750G	N35D	
	T1189C	I181T	
	A1438G	Ter264W	
MT-CO3	T9698C	*Syn*	
MT-ATP6	T8632C	Y36H	
	G9055A	A177T	
MT-ND6	C14167T	Syn	
MT-ND3	A10398G	T114A	
MT-ND4L	A10550G	*Syn*	
MT-TL2	A12308G	K15E	
MT-TD	A7559G	*Syn*	
Non-coding RNA	A73G		
	A263G		
	C497T		
	T16093C		
	T16224C		
	T16311C		
	G16390A		
	T16519C		

Table S4. Significant differences in mutation context counts between heated *vs.* control DNA of human breast normal cells determined using SSCS analysis. Significant *p* values are denoted with * (<0.05), ** (<$5x10^{-4}$), and *** (<$5x10^{-5}$) by the Chi-square test.

**C>A**	**C>G**	**C>T**	**T>A**	**T>C**	**T>G**
ACA *	CCA ***	ACT *	ATC *	ATT *	
ACC *	CCC ***	CCT **	ATG *	GTC *	
ACT **	CCG *	TCG *	ATT *	GTG *	
CCA *	CCT **		CTA *	TTA *	
CCG *	GCA *		CTC *	TTT *	
GCT *	TCA ***		GTA ***		
	TCC ***		TTA ***		

Table S5. Sequencing data yield of human breast normal cells (I and II).

Cells	Avg SSCS depth	Total sequenced SSCS Nts	**SSCS: No. of rare muts**	**SSCS: Rare mut freq**	Avg DCS depth	Total sequenced DCS Nts	**DCS: No. of rare muts**	**DCS: Rare mut freq**
II (Data for Figs 5, 6, S5, S6)	11622	192572847	**15429**	**8.01 $\times 10^{-5}$**	2510	41582269	**635**	**1.53 $\times 10^{-5}$**
I (Data for Figs S5, S6)	11045	183007737	**14881**	**8.13 $\times 10^{-5}$**	2460	40754407	**571**	**1.40 $\times 10^{-5}$**

Abbreviations used are: Avg, average; Nts, nucleotides; SSCS, single strand consensus sequences; DCS, duplex consensus sequences; mt, mitochondria; mut, mutation; freq, frequency

Table S7. Heat-induced artifactual rare variants identified for the thirteen protein-coding mtDNA genes of human breast normal cells. Variants were counted only once at each position of the genome. The percent variant of each gene was calculated by dividing number of variants observed by the DNA base length of the corresponding gene.

**Protein-coding genes**	**Gene base length**	**No. of variants**	**% variant of each gene**
MT-ND5	1812	379	20.92
MT-CO1	1542	299	19.39
MT-ND4	1378	295	21.41
MT-CYB	1141	249	21.82
MT-ND2	1042	209	20.06
MT-ND1	956	168	17.57
MT-CO3	784	178	22.70
**MT-CO2**	684	160	**23.39**
MT-ATP6	681	129	18.94
MT-ND6	525	106	20.19
**MT-ND3**	346	45	**13.01**
MT-ND4L	297	66	22.22
MT-ATP8	207	45	21.74

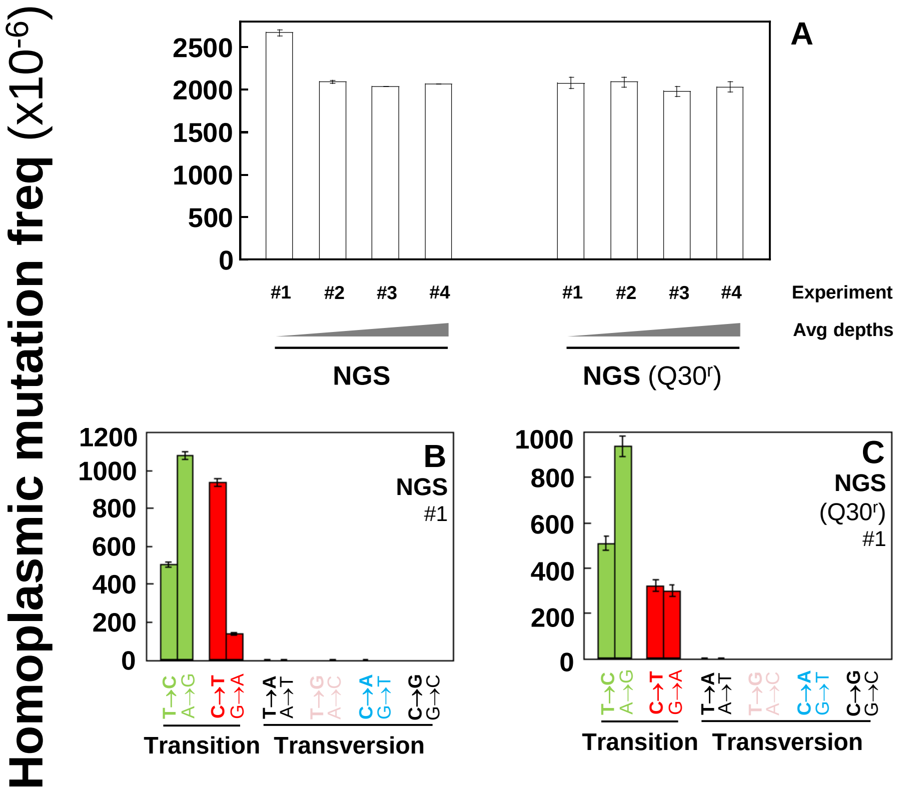

Figure S1. Frequencies of homoplasmic point mutations in the whole mtDNA of immortalized human breast cells. Error bars represent the Wilson score 95% confidence intervals. (**A**) Overall frequencies determined by performing conventional NGS before and after (Q30^r) bioinformatical modifications. The modifications include an increased base quality score of 30 (Q30) from the default score of 13 (Q13) and removal of PCR duplicates. Homoplasmic mutation frequency of specific mutation types determined using conventional NGS analysis before (**B**) and after (**C**) bioinformatical modifications.

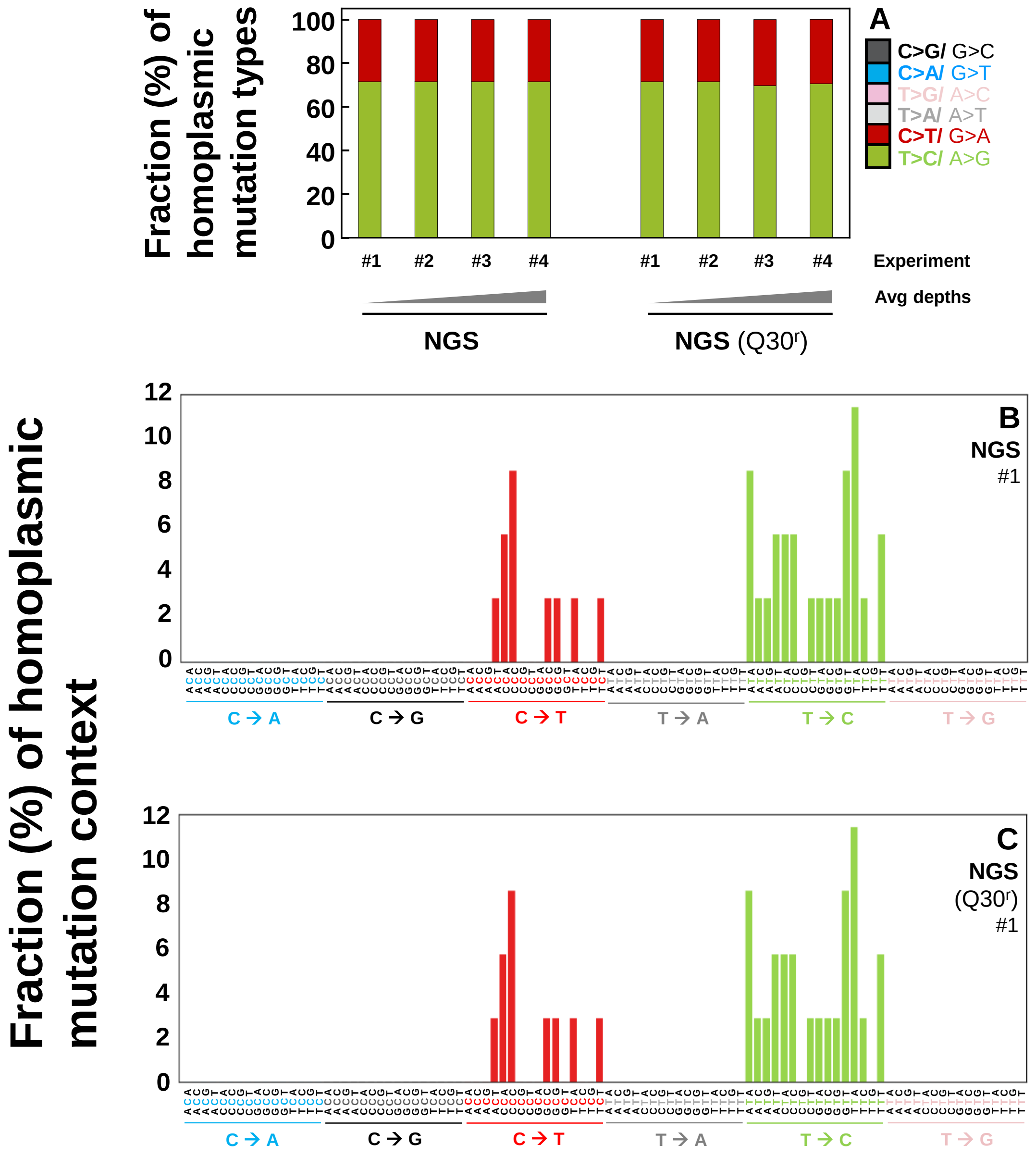

Figure S2. Fractions (%) of homoplasmic point mutation types and context spectra in the whole mtDNA of immortalized human breast cells. Relative percentages of mutation types (**A**) and fractions of rare mutation context spectra (**B,C**) were determined by performing conventional NGS analysis before and after (Q30^r) bioinformatical modifications. The modifications include an increased base quality score of 30 (Q30) from the default score of 13 (Q13) and removal of PCR duplicates.

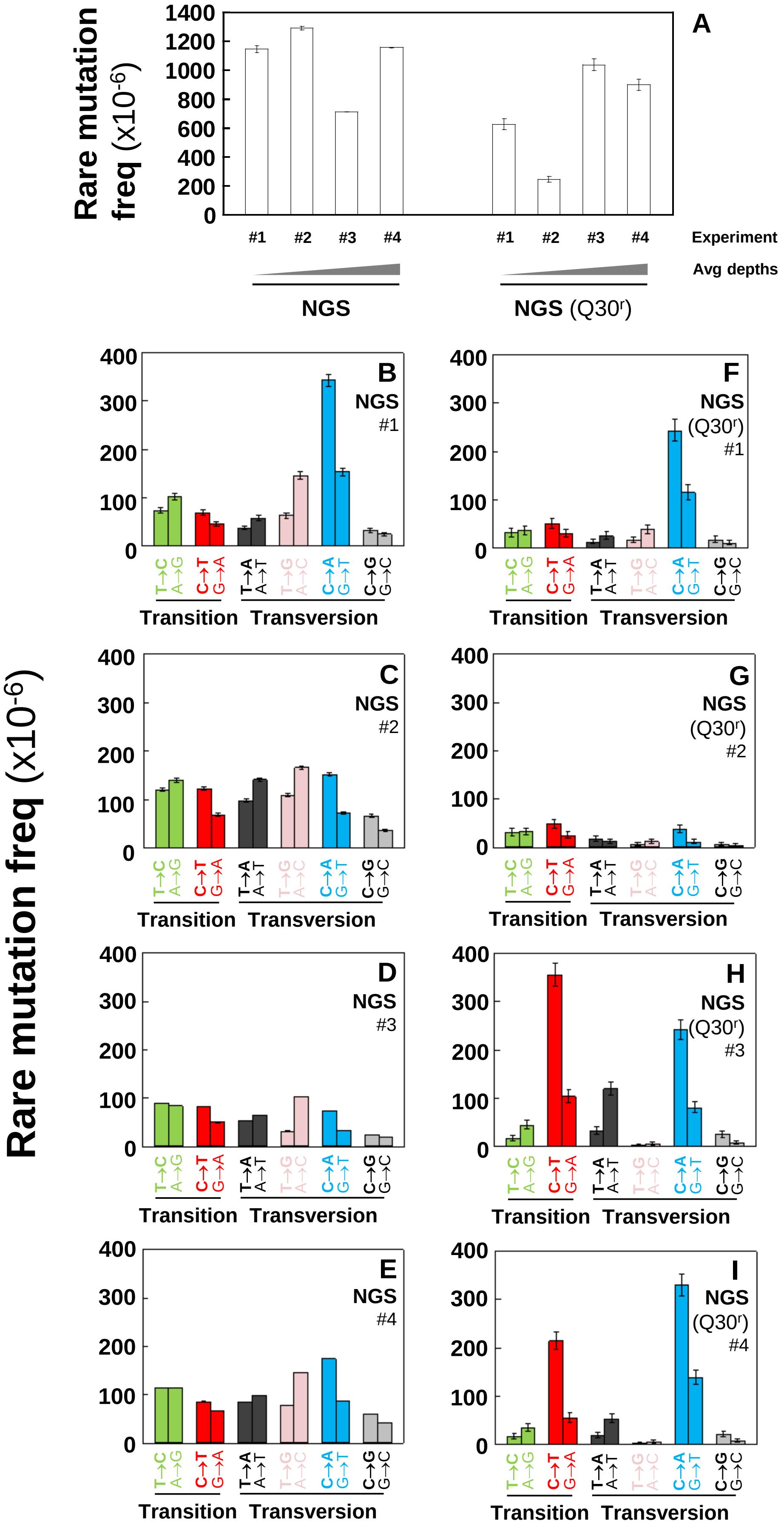

Figure S3. Frequencies of rare point mutations in the whole mtDNA of immortalized human breast cells. Error bars represent the Wilson score 95% confidence intervals. (**A**) Overall frequencies determined by performing conventional NGS analysis before and after (Q30r) bioinformatical modifications. The modifications include an increased base quality score of 30 (Q30) from the default score of 13 (Q13) and removal of PCR duplicates. Rare mutation frequency of each mutation type (**B-I**) as determined using conventional NGS analysis before and after (Q30r) bioinformatical modifications.

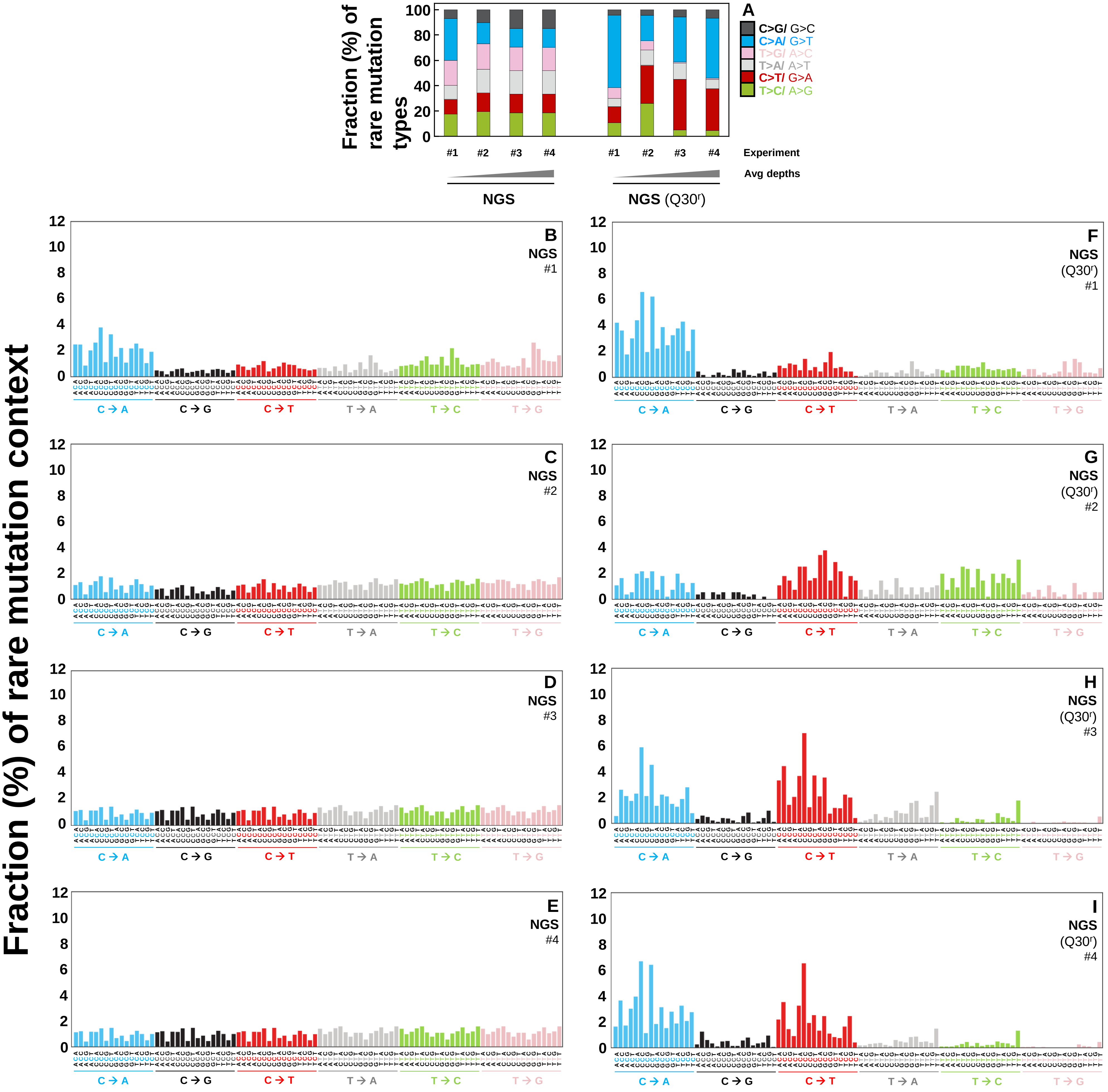

Figure S4. Fractions (%) of rare mutation types and context spectra in the whole mtDNA of immortalized human breast cells. Relative percentages of mutation types (**A**) and fractions of rare mutation context spectra (**B-I**) were determined by performing conventional NGS analysis before and after (Q30ʳ) bioinformatical modifications. The modifications include an increased base quality score of 30 (Q30) from the default score of 13 (Q13) and removal of PCR duplicates.

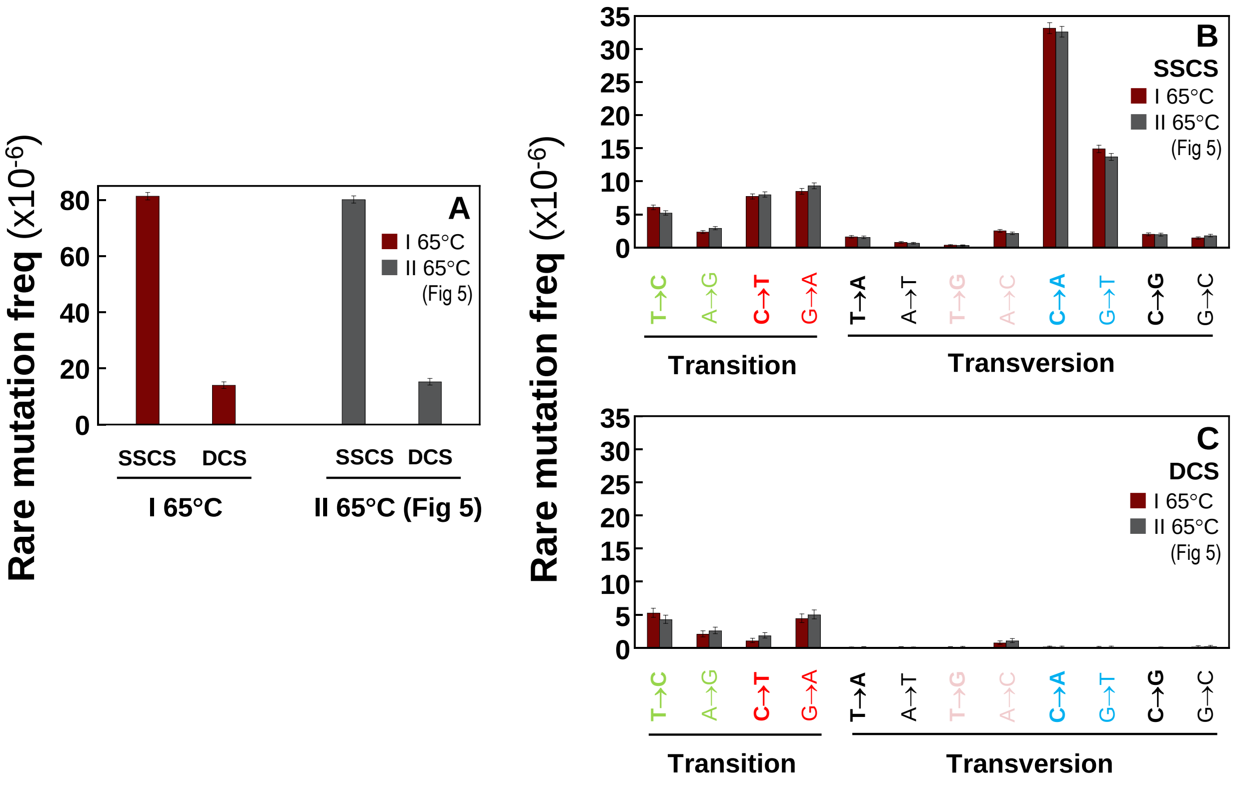

Figure S5. Frequencies of the heat-induced (65°C) artifactual rare variants in normal human breast cells I and II derived from breast tissue of the same woman. Data were generated by conducting two independent DNA library experiments (I and II). The purified DNA were heated at 65°C as described for Fig 5 and Fig 6. Overall rare mutation frequency (**A**) and frequencies of rare mutation types (**B,C**) for experiments I and II were determined by SSCS and DCS analyses. Error bars represent the Wilson score 95% confidence intervals.

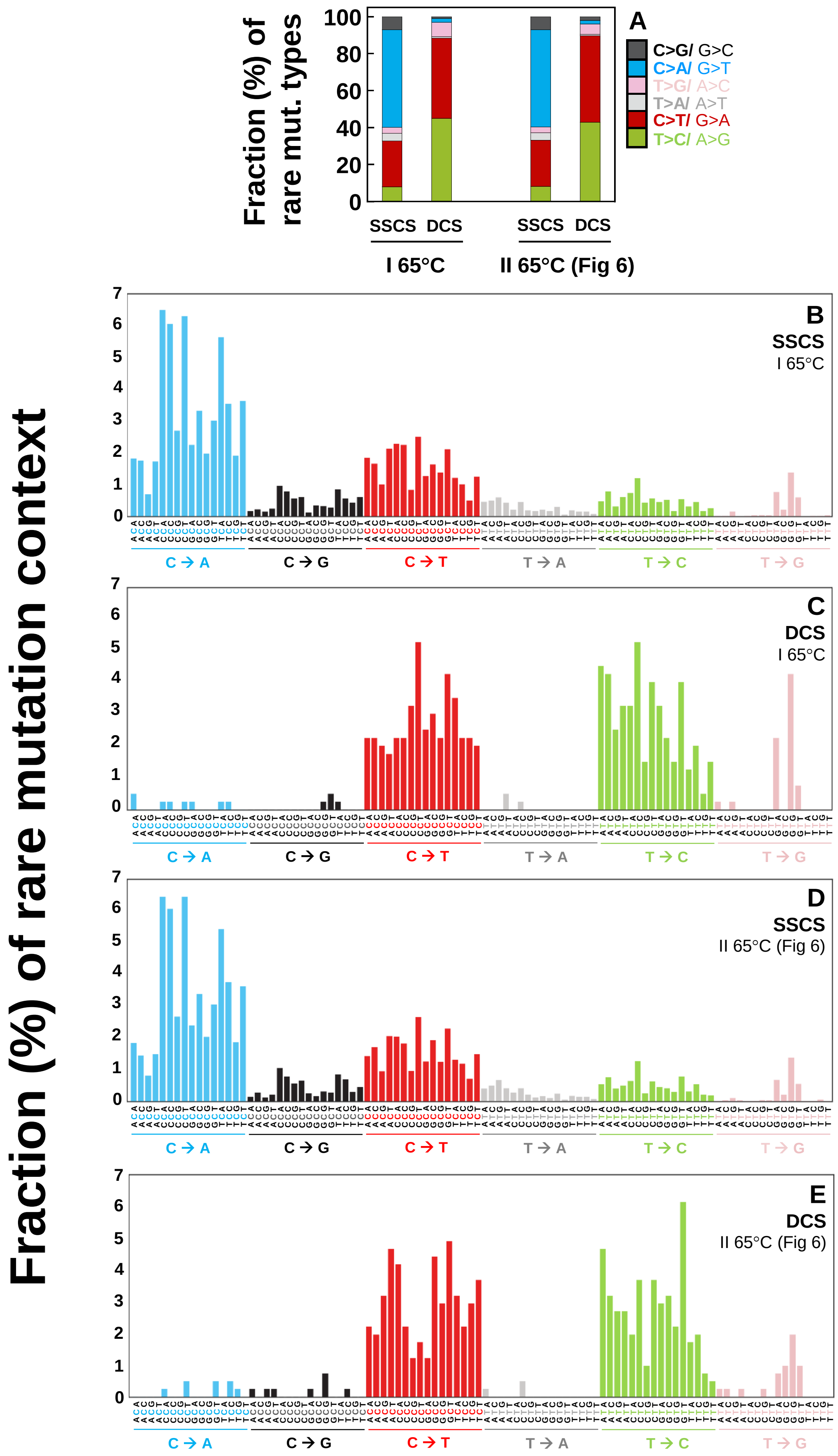

Figure S6. Fractions (%) of the heat-induced (65°C) artifactual rare mutation types and context spectra in normal human breast cells I and II derived from breast tissue of the same woman. Data were generated by conducting two independent DNA library experiments (I and II). The purified DNA was heated at 65°C as described for Fig 5 and Fig 6. Relative percentages of rare mutation types (**A**) and rare mutation context spectra (**B-E**) for experiments I and II were determined by SSCS and DCS analyses.
